# Beneficial Effects of Coculturing Synovial Derived Mesenchymal Stem Cells with Meniscus Fibrochondrocytes Are Mediated by Fibroblast Growth Factor 1: Increased Proliferation and Collagen Synthesis

**DOI:** 10.1155/2015/926325

**Published:** 2015-03-16

**Authors:** Xuanhe Song, Yaoping Xie, Yang Liu, Ming Shao, Wenbo Wang

**Affiliations:** ^1^Department of Orthopaedic Surgery, The First Affiliated Hospital of Harbin Medical University, Harbin, Heilongjiang 150001, China; ^2^Department of Nuclear Medicine, The First Affiliated Hospital of Harbin Medical University, Harbin, Heilongjiang 150001, China

## Abstract

Meniscus reconstruction is in great need for orthopedic surgeons. Meniscal fibrochondrocytes transplantation was proposed to regenerate functional meniscus, with limited donor supply. We hypothesized that coculture of synovial mesenchymal stem cells (SSC) with meniscal fibrochondrocytes (me-CH) can support matrix production of me-CH, thus reducing the number of me-CH needed for meniscus reconstruction. A pellet coculture system of human SSC and me-CH was used in this study. Enhanced glycosaminoglycans (GAG) in coculture pellets were demonstrated by Alcian blue staining and GAG quantification, when compared to monoculture. More collagen synthesis was shown in coculture pellets by hydroxyproline assay. Increased proliferation of me-CH was observed in coculture. Data from BrdU staining and ELISA demonstrated that conditioned medium of SSCs enhanced the proliferation and collagen synthesis of me-CH, and this effect was blocked by neutralizing antibody against fibroblast growth factor 1 (FGF1). Western blot showed that conditioned medium of SSCs can activate mitogen-activated protein kinase (MAPK) signaling pathways by increasing the phosphorylation of mitogen-activated regulated protein kinase 1/2 (MEK) and extracellular-signal-regulated kinases 1/2 (ERK). Overall, this study provided evidence that synovial MSCs can support proliferation and collagen synthesis of fibrochondrocytes, by secreting FGF1. Coimplantation of SSC and me-CH could be a useful strategy for reconstructing meniscus.

## 1. Introduction

Meniscus regeneration is a big challenge for orthopedic surgeons. In the past few decades, treatment of meniscus injury has been evolving tremendously since a number of new techniques had been invented [1]. Traditionally, operation is not necessary in many cases of patients with meniscal lesions. However, in symptomatic patients, a partial meniscectomy is suggested even sometimes with unstable outcomes [2]. Unlike articular cartilage which has limited potential of self-repair, meniscus shows some indications of self-healing due to its partial vascularization [3]. Based on this rationale, several new techniques, either simple, such as needling, abrasion, trephination, and gluing, or complex, such as synovial flaps, meniscal wrapping, and the application of fibrin clots, have been applied to patients [4, 5]. Although these repair methods show efficacy to some extent, failures do occur in many cases. Developing new surgical procedure with the strategy of regenerative medicine has been proposed in recent years [6]. Regenerating functional and stable meniscal fibrocartilage may be realized by a combination of stem cells, biocompatible scaffold, and proper growth factors, with most advanced technology in cell biology, biomaterial science, and bioengineering [7].

From clinical perspective, the best cell source for meniscus regeneration is the meniscus tissue from the meniscectomy surgery, since donor site morbidity is not of concern. It was first reported in 2001 that tissue from meniscectomised meniscus could give rise to a fibrochondrocytes population that can be expanded* in vitro* and seeded on scaffold, resulting in a tissue engineered meniscus [8]. In another attempt to regenerate meniscal tissue, more sophisticated scaffolds were designed to support meniscus derived fibrochondrocytes. Compared to previous study, the new combination of scaffold and cells resulted in better mechanical property that is closer to native meniscal tissue [9]. Despite the promising results from these papers, short of supply prevented the application of this cell type from a large scale of meniscal repair and regeneration. Moreover, many patients, who need meniscal cell transplantation, have already experienced total meniscectomy. So, alternative cell sources for meniscus tissue engineering are in demand.

One alternative cell source for meniscus regeneration is mesenchymal stem cells (MSCs) isolated from many tissue. MSCs can be derived from many sources, including bone marrow [10], fat [11], umbilical cord [12], and pluripotent stem cells [13]. It is attractive for orthopedic surgeon because harvesting tissue in either open or arthroscopic surgery can be relatively easy [14]. It has first been reported by De Bari et al. in 2001 that a population of mesenchymal stem cells could be isolated from synovial membrane [15]. Since then, many researchers have reported the multipotent differentiation and* in vitro* expansion of synovium derived mesenchymal stem cells (SSCs), which lead to the great expectation of applying SSCs in cell-based therapy for muscle-skeleton diseases [16–18]. It is even believed that the SSCs are superior to mesenchymal stem cells (MSCs) derived from other sources of tissue in generating cartilage tissues [19]. It is reported that injection of synovial MSCs may help in the repair of meniscus by providing protection at the medial femoral articular cartilage in pig models of massive meniscal defect [20]. However, concern arises when negative results were reported on generating cartilaginous tissue with SSCs* in vivo* [21]. Instead of undergoing chondrogenic lineage, SSCs either died or support neoangiogenesis. These data suggested that SSC alone may not be sufficient to form good quality cartilaginous tissue. A combination of SSC and me-CH, however, could be a valuable cell source for meniscus tissue engineering and is worthy testing with newest lab techniques.

In this paper, we studied the influence of coculture of SSC and me-CHs on the proliferation and collagen synthesis of fibrochondrocytes and how the signal from SSC is mediated. Coculture system of human SSC and me-CH was applied. Our results provide important evidence for using mixture of SSC and me-CH as a cell source of meniscus regeneration.

## 2. Materials and Methods

### 2.1. Isolation of Human Fibrochondrocytes and Synovium Derived Mesenchymal Stem Cells

Meniscus and synovium of three human adult donors (all males; age 45–73 years) were obtained from the First Affiliated Hospital of Harbin Medical University within 24 hours postmortem by dissection of lateral or medial meniscus from knee joints of donors. Human fibrochondrocytes of meniscus (me-CH) were isolated by digesting tissue pieces for overnight in 1 mg/mL of collagenase type I (Worthington, Lakewood, NJ) dissolved in DMEM with 10% FBS, 100 U/mL penicillin, and 100 *μ*g/mL streptomycin. Human synovium derived mesenchymal stem cells (SSCs) were isolated according to procedures in previous publications [15]. Both me-CH and SSCs were cultured in proliferation medium (DMEM supplemented with 10% fetal bovine serum, 1% L-glutamine, 0.2 mM ascorbic acid, 100 U/mL penicillin, and 10 *μ*g/mL streptomycin) till passage 2.

The use of all human materials in this study has been approved by the Medical Ethical Committee of Harbin Medical University. All chemicals were purchased from Sigma-Aldrich (St. Louis, MO), unless otherwise stated.

### 2.2. Coculture of SSCs and me-CH in Pellets

For cocultures, 100,000 SSC and 100,000 me-CH cells were seeded in one well of 96-well plate with round bottom. The plate was centrifuged for 5 min at 500 ×g. For monocultures, 200,000 cells of SSCs or me-CH were seeded in the same way. Pellets were cultured in serum free medium (SF medium) containing DMEM supplemented with 1% L-glutamine, 0.2 mM ascorbic acid, 100 U/mL penicillin, and 10 *μ*g/mL streptomycin. Medium was refreshed twice a week.

### 2.3. Trilineage Differentiation

For adipogenic differentiation, SSCs were seeded at density of 12 000 cells/cm^2^ and cultured in adipogenic medium for 2 weeks. Fat droplets were stained by Oil Red O staining. For osteogenic differentiation, SSCs were seeded at density of 12 000 cells/cm^2^ and cultured in osteogenic medium (*α*-MEM supplemented with 10% FBS, 0.2 mM AsAP, 10-7 M of dexamethasone, and 5 mM of *β*-GP (*β*-glycerophosphate, 100 U penicillin/mL, and 100 *μ*g/mL streptomycin)) for 3 weeks. Mineralized nodules were visualized by Alizarin Red staining. For chondrogenic differentiation, 200 000 of cells were seeded per well in 96-well plate with round bottom. Cell pellets were made by centrifuge at 500 g for 5 min. Then pellets were cultured in chondrogenic differentiation medium (DMEM supplemented with 40 *μ*g/mL of proline, 50 *μ*g/mL ITS-premix, 50 *μ*g/mL of AsAP, 100 *μ*g/mL of sodium pyruvate, 10 ng/mL of TGF*β*3, 10-7 M of dexamethasone, 500 ng/mL of BMP6, 100 U penicillin/mL, and 100 *μ*g/mL streptomycin) for 3 weeks. Pellets were applied to histological examination as described in the following section.

### 2.4. Histology and Immunofluorescent Staining

Cell aggregates were fixed with 10% formalin for overnight, dehydrated with ethanol, and embedded in paraffin following standard procedures. A microtome (Leica, Bensheim, Germany) was used to cut 4 *μ*m thick sections. Slides were then deparaffinized with xylene, rehydrated with ethanol, and stained for sulfated glycosaminoglycans (GAG) with Alcian blue. Nuclei were counterstained with nuclear fast red. For immunofluorescent staining, 4 *μ*m thick sections were deparaffinized and rehydrated with standard protocol. Goat Anti-FGF1 antibody (R&D Systems, Minneapolis, MN) were incubated with sections overnight at 4°C. Then positively stained area was visualized with Alexa-594 conjugated Donkey Anti-Goat IgG (Abcam, Cambridge, MA). Nuclei were counterstained with DAPI (diamidino-2-phenylindole, Thermo Scientific, Rockford, IL).

### 2.5. GAG and DNA Quantification

Five aggregates from each group were washed with phosphate-buffered saline (PBS) and digested in proteinase K solution (1 mg/mL in Tris/EDTA buffer) for overnight at 56°C. GAG content was determined with 1,9-dimethylmethylene blue chloride (DMMB) staining by using an Multiskan GO Microplate Spectrophotometer (Thermo Scientific, USA) at an absorbance of 520 nm. Standard curve was made by a series of dilutions of chondroitin sulfate. Quantification of total DNA using a CyQUANT DNA Kit (Molecular Probes, Eugene, OR) was performed for normalization purpose.

### 2.6. Hydroxyproline Assay

Collagen content of the aggregate was assessed using the hydroxyproline assay, using previously described method [22]. Briefly, cell aggregates were digested in papain buffer (0.5 mg/mL of papain dissolved in 0.1 M Na_2_HPO_4_, 5 mM EDTA, and 5 mM L-Cysteine HCl) for overnight. The digested solutes were hydrolyzed in 6N HCl at 110°C for overnight. Hydroxyproline was then assayed spectrophotometrically at 560 nm after reaction with 0.05 M of chloramine-T and 10% (w/v in 2-methoxyethanol) *ρ*-dimethylaminobenzaldehyde, using a Multiskan GO Microplate Spectrophotometer (Thermo Scientific, USA). A standard curve was generated with L-hydroxyproline for calculating the hydroxyproline concentration.

### 2.7. Cell Tracking with Organic Fluorescent Dyes

The PKH67 Green Fluorescent Cell Linker (Sigma-Aldrich, St. Louis, MO) was used to track me-CH in cocultures pellets. Fibrochondrocytes were labeled according to the manufacturer's protocol. Briefly, cells were trypsinized and resuspended in PBS at a concentration of 2 × 10^6^ cells/mL. The cells were incubated with 4 *μ*M of PKH67 at 37°C for 5 minutes followed by an incubation at 4°C for 15 minutes. Cells were washed twice with PBS before applying to coculture experiments.

### 2.8. Bromodeoxyuridine (BrdU) Labeling and Staining

Proliferating cells in aggregates were labeled with BrdU and then stained with sheep polyclonal Anti-BrdU antibody (Abcam, Cambridge, MA) and Alexa-594 conjugated Donkey Anti-Sheep IgG (Abcam, Cambridge, MA). Briefly, cell aggregates were cultured in proliferation differentiation medium containing 10 *μ*M of BrdU for 24 hours before cryosection. 10 *μ*m sections were cut with a cryotome (Leica, Bensheim, Germany). Sections were used for BrdU with primary and secondary antibodies. Nuclei were counterstained with DAPI (Thermo Scientific, Rockford, IL).

### 2.9. Collection of Conditioned Medium

For conditioned medium (Con medium), DMEM was incubated with SSCs of 90% confluence for 48 h, passed through a 0.22 mm filter, put in Amicon Ultra-15 Centrifugal Filter Unites (Millipore, Billerica, MA) with a cut-off of 3000 dalton Nominal Molecular Weight Limit, and centrifuged at 4000 ×g for 40 minutes.

### 2.10. Image Acquisition and Analysis

All images were made with an Eclipse Ti-E fluorescent microscope (Nikon, Tokyo, Japan). For coculture pellets, three images of each section were made to represent total cells (DAPI as blue), proliferating cells (BrdU as red), and me-CH (PKH67 as green). Then, ImageJ software [23] was used for cell counting. Briefly, we first set a threshold to avoid artifacts manually. Then we counted the number of green cells, red cells, green + red cells, and total cells by running plug-ins written with macro language of ImageJ. The percentage of BrdU positive me-CH in coculture pellets equals the number of green + red cells divided by the number of green cells multiplied by 100%. For monoculture pellets in SF medium or Con medium, two images of each section were made to represent total cells (DAPI as blue) and proliferating cells (BrdU as red). The percentage of BrdU positive me-CH in monoculture pellets equals the number of green cells divided by the number of total cells multiplied by 100%. At least 3 sections of each pellet and 2 pellets from each donor pair were imaged, quantified, and averaged. Values represent the mean ± standard deviation of at least 3 donors.

### 2.11. ELISA Assay

Two pellets from each donor of me-CH were digested with hyaluronidase (0.5 mg/mL, Worthington, Lakewood, NJ) and pepsin A (250 *μ*g/mL, Worthington, Lakewood, NJ) subsequently to extract collagens. Then, human type I collagen detection kit and human type II collagen detection kit (Chondrex Inc., Redmond, WA) was used to determine the amount of type I collagen and type II collagen according to the manufacturer's instructions. Absorbance was measured on Multiskan GO Microplate Spectrophotometer (Thermo Scientific, USA) at 450 nm. The absorbent value at 550 was also measured to subtract the absorbent value at 450 nm for correction of the optical imperfections in the microplates.

### 2.12. RNA Isolation and Quantitative PCR

RNA samples of cell pellets were isolated with the Mini RNA isolation Kit (Watson, Shanghai, China). Total RNA was reverse-transcribed into cDNA using the Maxima cDNA Synthesis kit (Thermo Scientific, San Diego, CA). Real-time quantitative PCR (qPCR) was performed on genomic DNA or cDNA samples by using the iQ SYBR Green Supermix (Bio-Rad, Hercules, CA). PCR reactions were carried out on MyiQ2 Two-Color Real-Time PCR Detection System (Bio-Rad, Hercules, CA). Melting curves were generated to test primer dimer formation and nonspecific priming. The sequences of primers for real-time PCR were obtained from primer bank of Harvard University (http://pga.mgh.harvard.edu/primerbank/). Sequences of primers used in this study are listed in Table 1. Calculations of relative expression (qPCR on cDNA) and relative amount (qPCR on genomic DNA) were performed with the double delta Ct method [24].

### 2.13. Protein Extraction and Western Blot

Cells were washed three times with PBS; then total proteins were extracted by protein extraction reagents (Thermo Scientific, Rockford, IL), followed by centrifugation (13,000 g for 15 min.) to remove cellular debris. Protein concentration was assessed by Pierce BCA Protein Assay Kit (Thermo Scientific, Rockford, IL). Twenty-five microgram of total proteins was analyzed by Western blotting using polyclonal rabbit phospho-MEK 1/2 (Ser217/221) antibody, MEK1/2 antibody, phospho-Erk1/2 (Thr202/Tyr204) antibody, or Erk1/2 antibody. All primary antibodies for Western blot were purchased from Cell Signaling Technologist (Danvers, MA). HRP (horseradish peroxidase) labeled secondary antibody against rabbit IgG (Abcam, Cambridge, MA). Immunocomplexes were visualized using enhanced chemiluminescence reagent (Thermo Scientific, Rockford, IL) according to the manufacturer's instructions.

### 2.14. Statistical Analysis

Both one-way analysis of variance (ANOVA) and Student's *t*-test were used for statistical analysis. Method for individual experiment was indicated in figures' legends. *P* values of < 0.05 were considered significant.

## 3. Results

### 3.1. Coculture Increases GAG Deposition and Collagen Synthesis

**Figure 1:**
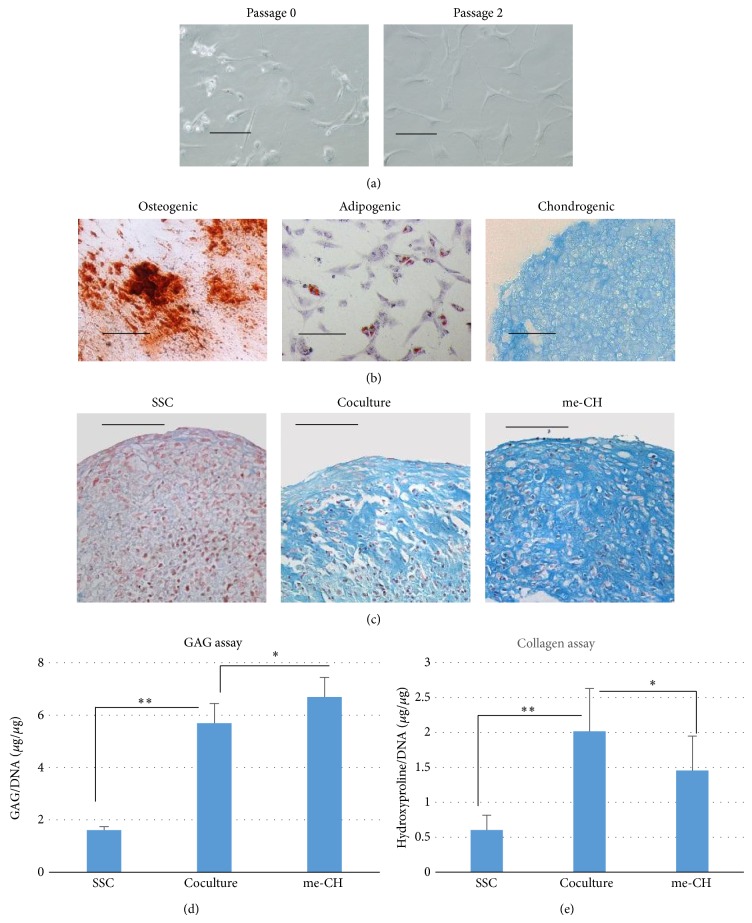
Coculture of SSCs and me-CH increases GAG formation and collagen biosynthesis. (a) Morphology of me-CH at passage 0 and passage 2. (b) Multilineage differentiation assay shows that SSCs are able to differentiate into osteoblast (osteogenic), adipocyte (adipogenic), and chondrocytes (chondrogenic). One representative donor is shown. (c) Alcian blue staining was performed at week 4 to detect GAGs. Scale bar = 100 *μ*m. (d) Quantitative GAG assay shows more GAGs deposited in coculture aggregates than monocultures of either SSC or me-CH (*n* = 5) at week 4. Error bar reflects standard deviation. (e) Total collagen contents were measured by hydroxyproline assay. The amount of hydroxyproline was expressed as *μ*g/*μ*g of DNA. ^*^ *P* < 0.05. ^**^ *P* < 0.01. *P* values were calculated with one-way ANOVA followed by Dunnett's test.

Upon isolation from meniscus, fibrochondrocytes showed two types of morphology. Some of them are round-shaped, while others are fibroblast-like. After a few passages, a homogenous morphology appears (Figure 1(a)). SSCs were then cultured in differentiation medium to test their multilineage differentiation potentials (Figure 1(b)). Passage 2 of me-CH was used to coculture with SSC (passage 2). Monoculture of me-CH or SSC was used as control. All cell pellets were cultured in SF medium (serum free medium containing 1% L-glutamine, 0.2 mM ascorbic acid, 100 U/mL penicillin, and 10 *μ*g/mL streptomycin). At week 4 after culture, pellets were harvested for histology, GAG quantification, and hydroxyproline assay. Alcian blue staining on paraffin sections showed evidence of sulfate GAG deposition in coculture and me-CH pellets (Figure 1(c)). Positively stained area retained typical morphology of fibrocartilage with chondrocytes emended long fibers. Meanwhile, barely any GAGs were detected in SSCs only pellets. As shown in Figure 1(d), GAGs in coculture pellets are about 5-fold more than the monoculture of SSC and 20% less than me-CH only pellets. This suggested that in coculture of two cells types, 50% of fibrochondrocytes produced 80% of GAGs compared to monoculture of me-CH in a 3-dimensional culture environment.

Then, total collagens were measured by hydroxyproline assay. Hydroxyproline is the unique amino acid present in collagen. Its level correlates tightly with the amount of total collagen content. After being normalized to DNA, coculture pellets contained about 3 times more collagens than monocultures of SSC and 1.5 times more collagen contents than monoculture of me-CH (Figure 1(e)).

### 3.2. Coculture Increases Proliferation

**Figure 2:**
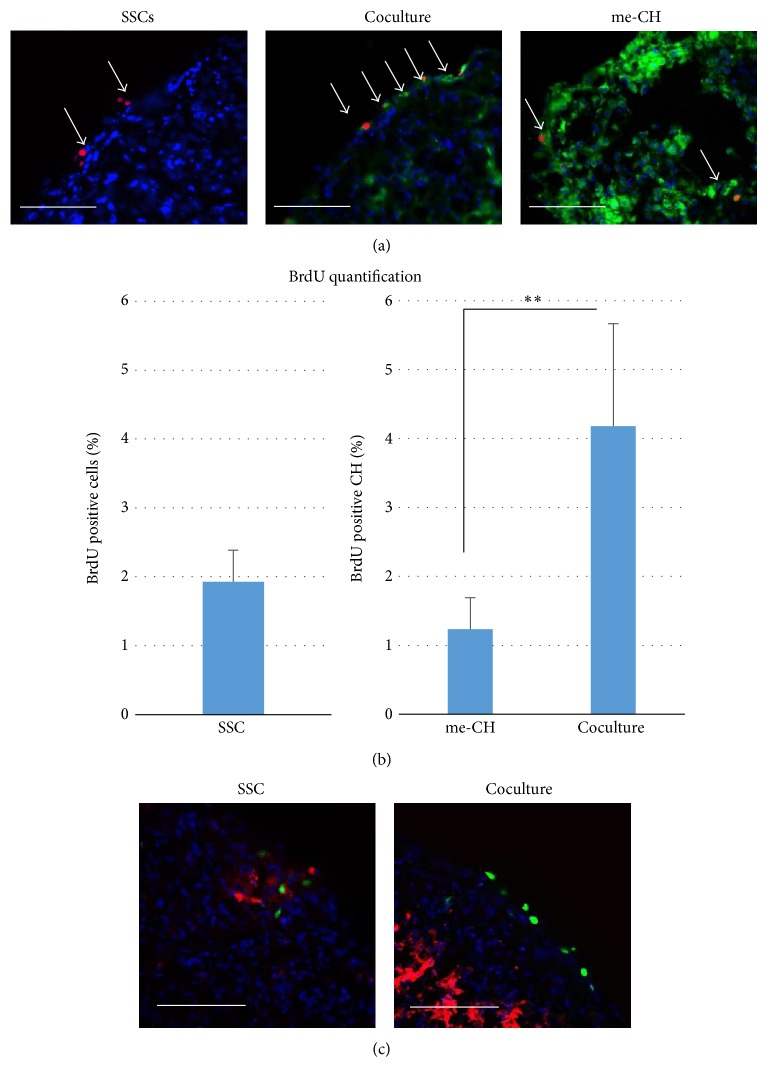
Coculture increases proliferation of me-CH. (a) BrdU was stained for proliferating cells at day 3. Positive cells are shown in red, indicated by white arrowheads. Green cells are PKH67 labeled me-CH. Nuclei were counterstained with DAPI (blue). Scale bar = 50 *μ*m. (b) BrdU positive cells were quantified (*N* = 3). Data is shown as mean + standard deviation. Statistical significance was analyzed by Student's *t*-test. ^**^ *P* < 0.01. (c) Immunofluorescent and BrdU staining for FGF1 is performed on SSCs pellet and coculture pellet. Red fluorescence shows positive staining of FGF1. Nuclei were counterstained with DAPI (blue). Scale bar = 50 *μ*m.

To test if coculturing with SSCs influences proliferation of me-CH, BrdU staining was combined with cell labeling with PKH67 fluorescent cell tracker. The ratio between me-CH and SSC in coculture is 1 : 1. Pellets of SSC or me-CH alone were used as controls. All pellets were cultured in SF medium. At day 3, BrdU was added to SF medium for incorporation. Twenty-four hours later, staining was performed to visualize BrdU. As shown in Figure 2(a), most BrdU positive cells in coculture aggregates are also green cells which are me-CH labeled with PKH67. Red and green cells were counted and divided by the number of green cells to calculate the percentage of BrdU positive me-CH in coculture pellets. Shown in Figure 2(b), more than 4% me-CH in coculture pellets are BrdU positive, while less than 2% in me-CH monoculture pellets are so. Difference between two groups is statistically significant (*P* < 0.001). Proliferating cells in SSC pellets were also quantified as control. Then, FGF1 staining was performed together with BrdU staining to examine the relationship between FGF1 expression and proliferation. FGF1 expression in SSC pellets is close to proliferating cells, while, in coculture pellets, FGF1 expression is underneath the surface layer of proliferating cells. More importantly, FGF1 expression in coculture pellets is much higher than in SSC pellets.

### 3.3. Conditioned Medium of SSCs Increases Proliferation of me-CH

**Figure 3:**
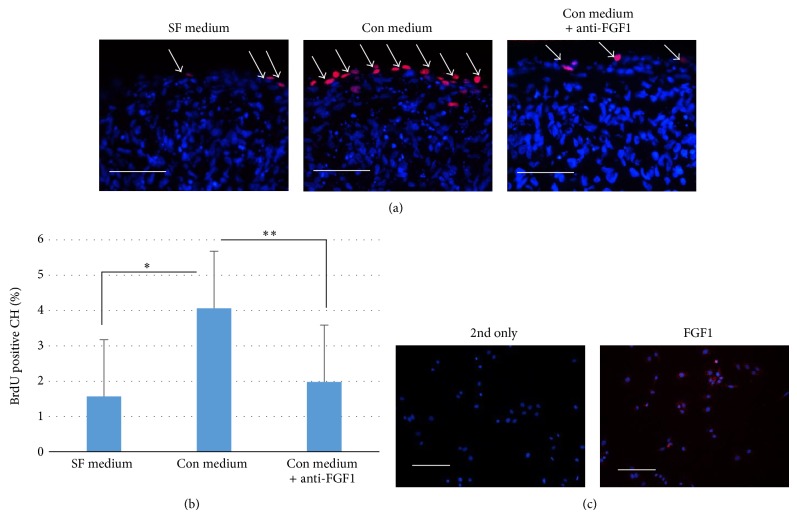
Conditioned medium of SSCs increases proliferation of me-CH through FGF1 signaling pathway. (a) BrdU was stained for proliferating cells at day 3 after forming of aggregates. Pellets of me-CH were cultured in SF medium (serum free medium plus 5 *μ*g/mL normal goat IgG), Con medium (conditioned medium plus 5 *μ*g/mL normal goat IgG), or Con medium + anti-FGF1 (conditioned medium plus 5 *μ*g/mL of goat antibody against FGF1). Positive cells are shown in red, indicated by white arrowheads. Nuclei were counterstained with DAPI (blue). Scale bar = 50 *μ*m. (b) BrdU positive cells were quantified (*N* = 3). Data is shown as mean + standard deviation. Statistical significance was analyzed by one-way ANOVA followed by Dunnett's test. ^*^ *P* < 0.05. ^**^ *P* < 0.01. (c) Immunofluorescent staining for FGF1 is performed on SSCs (passage 2). Red fluorescence shows positive staining of FGF1. Nuclei were counterstained with DAPI (blue). Scale bar = 50 *μ*m.

To study the mechanism how SSCs increase proliferation of me-CH in coculture, conditioned medium of SSCs was collected and used to culture me-CH pellets. An FGF1 neutralizing antibody (5 *μ*g/mL) was added to Con medium to neutralize active FGF1, since it is reported that FGF1 can be secreted by mesenchymal stem cells to stimulate the proliferation of chondrocytes [25]. Normal goat IgG (5 *μ*g/mL) was added to SF medium and Con medium to eliminate the effects of unspecific binding of IgG. As shown in Figure 3(a), very few me-CH are BrdU positive, while much more positive cells were seen on the periphery of pellets cultured in Con medium. Stimulatory effects of Con medium on me-CH disappeared after neutralization of anti-FGF1 antibody. Quantification of BrdU positive cells in all conditions confirmed our impression on the proliferating cells (Figure 3(b)). Increase of BrdU positive cells in Con medium is significant when compared to SF medium and Con medium plus anti-FGF1. Expression of FGF1 by SSCs was evidenced by immunofluorescent staining on passage 2 cells (Figure 3(c)).

### 3.4. Conditioned Medium of SSCs Increases Collagen Synthesis of me-CH

**Figure 4:**
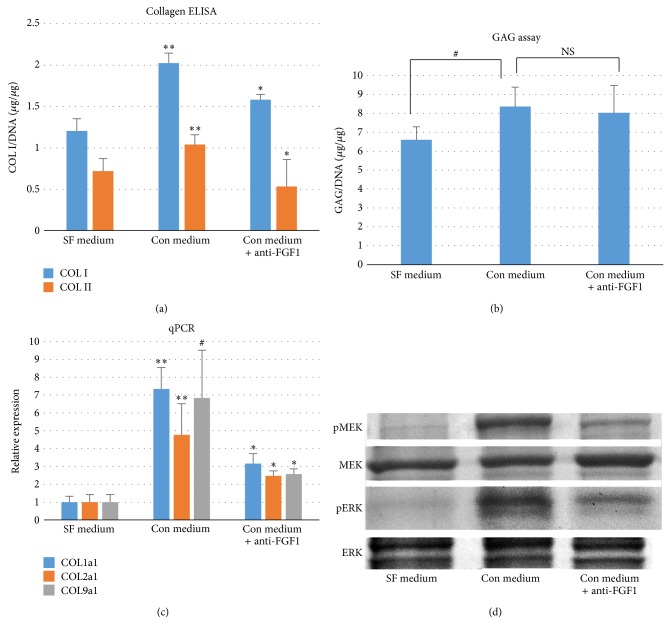
Increased collagen synthesis of me-CH by conditioned medium of SSCs is mediated by FGF1. (a) ELISA was performed to measure collagen type I (a) or collagen type II (b) in me-CH cultured in SF medium (serum free medium plus 5 *μ*g/mL normal goat IgG), Con medium (conditioned medium plus 5 *μ*g/mL normal goat IgG), or Con medium + anti-FGF1 (conditioned medium plus 5 *μ*g/mL of goat antibody against FGF1). Collagen amount was normalized to total DNA in pellets (*N* = 3). *P* values were calculated with one-way ANOVA followed by Dunnett's test. (b) Quantitative GAG assay (*n* = 3). Error bar reflects standard deviation. NS: nonsignificance. (c) Expressions of three collagens (1a1, 2a1, and 9a1) were measured by qPCR in me-CH pellets cultured in the same three conditions. GAPDH was used for normalization. me-CH in SF medium was chosen as reference. Number in coculture represents the relative expression level of corresponding gene compared to me-CH. Three donor pairs were analyzed. (d) Total protein was extracted from me-CH cultured in the same three conditions for 1 hour in 2D environment. Western blot was then performed to detect the phosphorylation of MEK1/2 and ERK1/2. Total MEK1/2 and ERK1/2 were shown as controls. ^**^ *P* < 0.05, when comparing Con medium group with SF medium group. ^*^ *P* < 0.01, when comparing Con medium + anti-FGF1 group with Con medium group. ^#^ *P* < 0.05, when comparing Con medium group with SF medium group.

To test if SSCs increase collagen synthesis of me-CH by secreting FGF1, collagen type I and type II were quantified by ELISA. Pellets of me-CH cultured in SF medium plus normal goat IgG (5 *μ*g/mL) served as control. One group of pellets were cultured in Con medium plus normal goat IgG (5 *μ*g/mL). Another group of pellets were cultured in Con medium plus FGF1 neutralizing antibody (5 *μ*g/mL). Data from both collagen type I and type II quantifications showed an increased in Con medium group compared to control group (Figure 4(a)). Neutralizing activity of FGF1 diminished the effects of Con medium. Results of GAG showed that conditioned medium of SSCs increases GAG of me-CH; however, this effect was not reversed by adding antibody of FGF1 in the conditioned medium (Figure 4(b)). To validate the increase of collagen expressions at mRNA level, real-time qPCR was performed. Expressions of 3 collagen genes were found to be higher in pellets cultured in Con medium than those in SF medium or Con medium plus anti-FGF1 antibody (Figure 4(c)). More specifically, expressions of COL1a1 and COL9a1 are almost 7 times higher in Con medium than in SF medium, while COL2a1 is expressed about 4 times more in Con medium compared to SF medium. To further confirm the signaling pathways activated by Con medium, Western blot was performed to test the phosphorylation of MEK1/2 and ERK1/2. As shown in Figure 4(d), Con medium increases phosphorylation of both MEK1/2 and ERK1/2, while neutralizing FGF1 in Con medium can reverse the phosphorylation of MAP kinases.

## 4. Discussion

MSCs are believed to be recruited to the injury site and to participate in the natural healing process of injured tissue [26]. Not only providing necessary cell sources for tissue regeneration but also secreting trophic factors that support tissue repair, these features of MSCs make them attractive for tissue engineering and regenerative medicine [27]. Particularly the trophic effects of MSCs shown in the process of healing are extremely valuable for regenerating tissues that have limited capacity of self-repair, such as cartilage [28]. Recently, it has been reported that MSCs benefited cartilage formation in coculture with chondrocytes by secreting trophic factors that supported the proliferation and matrix deposition of chondrocytes but not actively underwent chondrogenic differentiation [29, 30]. MSCs derived from synovium membrane were shown to have similar trophic effects as bone marrow and fat derived MSCs in supporting chondrocytes proliferation and GAG production [31]. With some debates going on, similar results were obtained by other research groups [32–34].

As summarized by a more recent review article [35], the supportive effects of MSCs in cartilage regeneration can be divided into three parts: first, MSCs promoted GAG production of chondrocytes; second, MSCs increase proliferation of chondrocytes; third, MSCs disappear in coculture with chondrocytes. In this study, our data demonstrated that trophic effects of MSCs not only work on articular chondrocytes but also on fibrochondrocytes. We believe that it is an important supplement to the current understanding of MSCs' role of trophic mediators in tissue regeneration.

More specifically, it has been reported that cocultures of primary meniscus cells and bone marrow derived MSCs resulted in more deposition of GAG comparing to single coculture of BM-MSCs or meniscus cells [36]. Similarly, the coculture of synovium derived stem cells (SDSC) with meniscus cells resulted better in cell survival and differentiation into chondrogenic lineage, as demonstrated by more glycosaminoglycan, collagen II, and Sox 9, but low collagen I [37]. These data are in line with our results obtained from SSCs. Beyond the observation in this study, our data also demonstrated that coculture increases collage synthesis at a protein level. In another relevant study, it is shown that both inner meniscus cells and outer meniscus cells deposit more GAGs when cocultured with MSCs [38]. Moreover, meniscus cells suppressed hypertrophic differentiation of MSCs, and outer cells seemed to be more effective than inner cells. In our experiment, we did not separate inner and outer menisci. It would be interesting to investigate the difference of cells isolated from multiple locations of meniscus in our system.

FGF1 is initially considered as a mitogen for endothelial cells [39, 40]. It is then reported to promote cell proliferation for many different cell types, including vascular smooth muscle, chondrocytes, osteoblasts, fibroblasts, and glial cells [41]. It belongs to FGF superfamily of 22 members in humans and mice [42]. Members from this family, for example, FGF2 and FGF18, were previously reported to benefit chondrogenic differentiation and cartilage repair [43, 44]. These FGFs are believed to play important role in normal physiological processes and pathological conditions [45]. By activating a group of FGF receptors (FGFRs) which are all tyrosine kinase cell-surface receptors, FGF1 induced extracellular signal-regulated kinase 1/2 (ERK1/2) signaling to stimulate the proliferation of the ligament-derived epithelial progenitor-like fibroblasts [46]. Our data is in line with these previous reports showing the FGF1 secreted from SSCs increased proliferation and collagen synthesis by activating MEK/ERK signaling pathways. By neutralizing FGF1 activity in conditioned medium of SSC, both proliferation and collagen synthesis can be suppressed in me-CH. Interestingly, our data showed that expression of FGF1 is upregulated in coculture pellets when compared to SSC pellets. This indicated that cellular interactions between SSC and fibrochondrocytes somehow increase expression of FGF1.

In conclusion, our data demonstrated that SSC supported proliferation, GAG formation, and collagen synthesis of fibrochondrocytes derived from meniscus. This study provides new evidence for the supportive effects of synovium derived mesenchymal stem cells played in cartilage regeneration, expanding the effects from articular chondrocytes to fibrochondrocytes. Supplementing meniscal cells with SSCs not only solves the problem of limited cell numbers from meniscus, but also helps meniscal cells to grow. Our findings suggest that coculture of the two types of cells could be used as cell source for engineering functional grafts to reconstruct meniscus.

## Figures and Tables

**Table 1:** Sequences for primers.

Gene name	NCBI gene ID	Sequence (5′ → 3′)	Length of amplicon
*COL1a1 *	1277	Forward: GAGGGCCAAGACGAAGACATC Reserve: CAGATCACGTCATCGCACAAC	140

*COL2a1 *	1280	Forward: TGGACGATCAGGCGAAACC Reserve: GCTGCGGATGCTCTCAATCT	244

*COL9a1 *	7474	Forward: GGCAGTAGAGGAGAATTAGGACC Reverse: GTTCACCGACTACACCCCTG	142

Glyceraldehyde-3-phosphate dehydrogenase (GAPDH)	2597	Forward: CTGGGCTACACTGAGCACC Reserve: AAGTGGTCGTTGAGGGCAATG	101

## References

[1] DeHaven K. E. (1990). Decision-making factors in the treatment of meniscus lesions. *Clinical Orthopaedics and Related Research*.

[2] Noble J., Turner P. G. (1986). The function, pathology, and surgery of the meniscus. *Clinical Orthopaedics and Related Research*.

[3] DeHaven K. E. (1985). Rationale for meniscus repair or excision. *Clinics in Sports Medicine*.

[4] Jacobi M., Jakob R. P. (2010). Meniscal repair: enhancement of healing process. *The Meniscus*.

[5] Scordino L. E., DeBerardino T. M. (2012). Biologic enhancement of meniscus repair. *Clinics in Sports Medicine*.

[6] Pereira H., Frias A. M., Oliveira J. M., Espregueira-Mendes J., Reis R. L. (2011). Tissue engineering and regenerative medicine strategies in meniscus lesions. *Arthroscopy*.

[7] Scotti C., Hirschmann M. T., Antinolfi P., Martin I., Peretti G. M. (2013). Meniscus repair and regeneration: review on current methods and research potential. *European Cells and Materials*.

[8] Nakata K., Shino K., Hamada M. (2001). Human meniscus cell: characterization of the primary culture and use for tissue engineering. *Clinical Orthopaedics and Related Research*.

[9] Baker B. M., Nathan A. S., Huffman G. R., Mauck R. L. (2009). Tissue engineering with meniscus cells derived from surgical debris. *Osteoarthritis and Cartilage*.

[10] Conget P. A., Minguell J. J. (1999). Phenotypical and functional properties of human bone marrow mesenchymal progenitor cells. *Journal of Cellular Physiology*.

[11] Zuk P. A., Zhu M., Mizuno H. (2001). Multilineage cells from human adipose tissue: implications for cell-based therapies. *Tissue Engineering*.

[12] Li N., Pan S., Zhu H., Mu H., Liu W., Hua J. (2014). BMP4 promotes SSEA-1^+^ hUC-MSC differentiation into male germ-like cells *in vitro*. *Cell Proliferation*.

[13] TheinHan W., Liu J., Tang M., Chen W., Cheng L., Xu H. H. (2013). Induced pluripotent stem cell-derived mesenchymal stem cell seeding on biofunctionalized calcium phosphate cements. *Bone Research*.

[14] Fan J., Varshney R. R., Ren L., Cai D., Wang D.-A. (2009). Synovium-derived mesenchymal stem cells: a new cell source for musculoskeletal regeneration. *Tissue Engineering, Part B: Reviews*.

[15] De Bari C., Dell'Accio F., Tylzanowski P., Luyten F. P. (2001). Multipotent mesenchymal stem cells from adult human synovial membrane. *Arthritis & Rheumatism*.

[16] Gimeno M. J., Maneiro E., Rendal E., Ramallal M., Sanjurjo L., Blanco F. J. (2005). Cell therapy: a therapeutic alternative to treat focal cartilage lesions. *Transplantation Proceedings*.

[17] de la Garza-Rodea A. S., van der Velde-van Dijke I., Boersma H. (2012). Myogenic properties of human mesenchymal stem cells derived from three different sources. *Cell Transplantation*.

[18] Miyamoto T., Muneta T., Tabuchi T. (2010). Intradiscal transplantation of synovial mesenchymal stem cells prevents intervertebral disc degeneration through suppression of matrix metalloproteinase-related genes in nucleus pulposus cells in rabbits. *Arthritis Research and Therapy*.

[19] Sakaguchi Y., Sekiya I., Yagishita K., Muneta T. (2005). Comparison of human stem cells derived from various mesenchymal tissues: superiority of synovium as a cell source. *Arthritis and Rheumatism*.

[20] Hatsushika D., Muneta T., Nakamura T. (2014). Repetitive allogeneic intraarticular injections of synovial mesenchymal stem cells promote meniscus regeneration in a porcine massive meniscus defect model. *Osteoarthritis and Cartilage*.

[21] de Bari C., Dell'Accio F., Luyten F. P. (2004). Failure of in vitro-differentiated mesenchymal stem cells from the synovial membrane to form ectopic stable cartilage in vivo. *Arthritis and Rheumatism*.

[22] Reddy G. K., Enwemeka C. S. (1996). A simplified method for the analysis of hydroxyproline in biological tissues. *Clinical Biochemistry*.

[23] Henriques R., Lelek M., Fornasiero E. F., Valtorta F., Zimmer C., Mhlanga M. M. (2010). QuickPALM: 3D real-time photoactivation nanoscopy image processing in ImageJ. *Nature Methods*.

[24] Livak K. J., Schmittgen T. D. (2001). Analysis of relative gene expression data using real-time quantitative PCR and the 2^-ΔΔCT^ method. *Methods*.

[25] Wu L., Leijten J., van Blitterswijk C. A., Karperien M. (2013). Fibroblast growth factor-1 is a mesenchymal stromal cell-secreted factor stimulating proliferation of osteoarthritic chondrocytes in co-culture. *Stem Cells and Development*.

[26] Caplan A. I., Dennis J. E. (2006). Mesenchymal stem cells as trophic mediators. *Journal of Cellular Biochemistry*.

[27] Caplan A. I. (2007). Adult mesenchymal stem cells for tissue engineering versus regenerative medicine. *Journal of Cellular Physiology*.

[28] Caplan A. I., Correa D. (2011). The MSC: an injury drugstore. *Cell Stem Cell*.

[29] Wu L., Leijten J. C. H., Georgi N., Post J. N., Van Blitterswijk C. A., Karperien M. (2011). Trophic effects of mesenchymal stem cells increase chondrocyte proliferation and matrix formation. *Tissue Engineering—Part A*.

[30] Acharya C., Adesida A., Zajac P. (2012). Enhanced chondrocyte proliferation and mesenchymal stromal cells chondrogenesis in coculture pellets mediate improved cartilage formation. *Journal of Cellular Physiology*.

[31] Wu L., Prins H.-J., Helder M. N., van Blitterswijk C. A., Karperien M. (2012). Trophic effects of mesenchymal stem cells in chondrocyte co-cultures are independent of culture conditions and cell sources. *Tissue Engineering, Part A*.

[32] Meretoja V. V., Dahlin R. L., Kasper F. K., Mikos A. G. (2012). Enhanced chondrogenesis in co-cultures with articular chondrocytes and mesenchymal stem cells. *Biomaterials*.

[33] Ko C. Y., Ku K. L., Yang S. R. (2013). In vitro and in vivo co-culture of chondrocytes and bone marrow stem cells in photocrosslinked PCL-PEG-PCL hydrogels enhances cartilage formation. *Journal of Tissue Engineering and Regenerative Medicine*.

[34] Levorson E. J., Santoro M., Kasper F. K., Mikos A. G. (2014). Direct and indirect co-culture of chondrocytes and mesenchymal stem cells for the generation of polymer/extracellular matrix hybrid constructs. *Acta Biomaterialia*.

[35] Wu L., Cai X., Zhang S., Karperien M., Lin Y. (2013). Regeneration of articular cartilage by adipose tissue derived mesenchymal stem cells: perspectives from stem cell biology and molecular medicine. *Journal of Cellular Physiology*.

[36] Matthies N.-F., Mulet-Sierra A., Jomha N. M., Adesida A. B. (2013). Matrix formation is enhanced in co-cultures of human meniscus cells with bone marrow stromal cells. *Journal of Tissue Engineering & Regenerative Medicine*.

[37] Tan Y., Zhang Y., Pei M. (2010). Meniscus reconstruction through coculturing meniscus cells with synovium-derived stem cells on small intestine submucosa—a pilot study to engineer meniscus tissue constructs. *Tissue Engineering, Part A*.

[38] Saliken D. J. J., Mulet-Sierra A., Jomha N. M., Adesida A. B. (2012). Decreased hypertrophic differentiation accompanies enhanced matrix formation in co-cultures of outer meniscus cells with bone marrow mesenchymal stromal cells. *Arthritis Research and Therapy*.

[39] Raman R., Venkataraman G., Ernst S., Sasisekharan V., Sasisekharan R. (2003). Structural specificity of heparin binding in the fibroblast growth factor family of proteins. *Proceedings of the National Academy of Sciences of the United States of America*.

[40] Burgess W. H., Maciag T. (1989). The heparin-binding (fibroblast) growth factor family of proteins. *Annual Review of Biochemistry*.

[41] Friesel R., Maciag T. (1999). Fibroblast growth factor prototype release and fibroblast growth factor receptor signaling. *Thrombosis and Haemostasis*.

[42] Itoh N., Ornitz D. M. (2004). Evolution of the Fgf and Fgfr gene families. *Trends in Genetics*.

[43] Hagmann S., Moradi B., Frank S. (2013). FGF-2 addition during expansion of human bone marrow-derived stromal cells alters MSC surface marker distribution and chondrogenic differentiation potential. *Cell Proliferation*.

[44] Correa D., Somoza R. A., Lin P. (2014). Sequential exposure to fibroblast growth factors (FGF) 2, 9 and 18 enhances hMSC chondrogenic differentiation. *Osteoarthritis and Cartilage*.

[45] Chen G. J., Forough R. (2006). Fibroblast growth factors, fibroblast growth factor receptors, diseases, and drugs. *Recent Patents on Cardiovascular Drug Discovery*.

[46] Takahashi M., Okubo N., Chosa N. (2012). Fibroblast growth factor-1-induced ERK1/2 signaling reciprocally regulates proliferation and smooth muscle cell differentiation of ligament-derived endothelial progenitor cell-like cells. *International Journal of Molecular Medicine*.

